# Tunable Wavelength-Multiplexed Dual-Frequency Bound Pulse in a Carbon-Nanotube-Based Fiber Laser

**DOI:** 10.3390/mi17010133

**Published:** 2026-01-20

**Authors:** Lin Wang, Guoqing Hu, Yan Wang, Guangwei Chen, Liang Xuan, Zhehai Zhou, Jun Yu

**Affiliations:** 1 East China Engineering Science & Technology Co., Ltd., Hefei 535019, China; wanglin@chinaecec.com (L.W.); wangyan@chinaecec.com (Y.W.); yujun@chinaecec.com (J.Y.); 2 Key Laboratory of Modern Optoelectronic Measurement Technology in Mechanical Industry, Beijing Information Science and Technology University, Beijing 100192, China; guoqing2011@foxmail.com (G.H.); zhouzhehai@bistu.edu.cn (Z.Z.)

**Keywords:** ultrafast fiber laser, dual-frequency solitons, bound states, coupled Ginzburg–Landau equations

## Abstract

We experimentally and theoretically demonstrate coexistence of three different wavelength-multiplexed bound dual-frequency pulses in an all-fiber mode-locked fiber laser, effectively achieved by exploiting polarization-dependent loss effects and two uneven gain peaks of Er-doped fiber. With the single wall carbon-nanotube-based intensity modulation, wavelength-multiplexed dual-frequency pulses located at 1531.1 nm and 1556.6 nm are obtained. Changing the polarization rotation angles in the fiber cavity, one of the two asynchronous pulses evolves into a bound state of a doublet, in which the center wavelength of the bound solitons is centered at ~1530 nm or ~1556 nm. The relative phase between the two bound solitons or modulation depth of bound solitons can be switched by a polarization controller. A simulation method based on coupled Ginzburg–Landau equations is provided to characterize the laser physics and understand the mechanism behind the dynamics of tuning between different bound dual-frequency pulses. The proposed fiber laser will provide a potential way to understand multiple soliton dynamics and implementation in optical frequency combs generation.

## 1. Introduction

Soliton fiber lasers have attracted much interest because of the rich passively mode locking dynamics and vital roles in photonics, biology and telecommunications [1,2,3,4,5,6]. By engineering the cavity parameters and intracavity devices, soliton dynamics behaviors can be manipulated, generating self-stabled soliton structures, such as bound solitons and periodic soliton pattern [7,8]. For example, the Tang group demonstrates bound states in a doublet and bunched solitons in a nonlinear polarization rotation (NPR)-based mode-locked fiber laser [9]. G. Herink et al. experimental investigated phase locked soliton molecules using real-time time-stretch technique, tracking the fast internal motion of bound states with different oscillating separation and phase [8]. The two solitons in a bunched pattern are randomly spaced in the fiber laser ring cavity, so various ways are implemented to arrange the solitons, such as introducing additive modulation, gain depletion, and harmonic mode-locked pulses [4,10,11]. As a special type of multiple pulse, dual-frequency solitons have been the focus of attention, because of the passive mutual coherence between pulses [12,13].

Wavelength-multiplexed fiber lasers are the common way to generate dual-frequency pulses. In order to obtain dual-frequency pulses, various optical filtering structures are introduced into the laser cavity. Li et al. have demonstrated a switchable dual-wavelength mode-locked pulses in all-polarization-maintaining fiber laser using a Sagnac loop filter [14]. Yun et al. presented a dual-wavelength soliton in a lead selenide quantum-dots-based fiber laser, with which the birefringence-induced spectral filtering effect leads to the generation of dual-frequency solitons [15]. Compared with these practical filters, the polarization-related gain profile is a simple and effective way of implementing the filtering effect. Mao et al. experimentally and numerically demonstrated the dual-wavelength operation in an NPR fiber laser, using the feature of dual-peak gain spectrum of the erbium-doped gain medium [16]. Li et al. used a section of polarization-maintaining fiber to generate polarization-multiplexed dual-frequency pulses, and a loss-based gain profile to obtain wavelength-multiplexed dual-frequency pulses [17]. To date, the research of single/multiple dimension multiplexed fiber lasers has focused on asynchronous pulse number, pulse width, spectral bandwidth, and repetition rate difference [18,19,20]. However, pulse type of wavelength-multiplexed fiber lasers have not been studied.

In this work, we experimentally and theoretically study a flexible tunable wavelength-multiplexed bound dual-frequency mode-locked fiber laser based on carbon nanotube (CNT). The tunable features can be obtained by utilizing polarization-dependent-loss and two gain peaks of Er-doped fiber. When the rotation angles are set as 46°, 67°, the spectral interferometry in two asynchronous dual-frequency pulses changes from a ~1530 nm pulse to a ~1556 nm pulse. When the rotation angle is 132°, the relative phase between the two bound solitons centered at ~1530 nm shifts π. These experimental results have been demonstrated by coupled Ginzburg–Landau equations. The simulation results indicate that the dual-frequency bound-pulse can be switched by changing the polarization rotations in the laser cavity. Our work will provide further understanding of multiple soliton interaction and optical frequency combs generation.

## 2. Methods

**Figure 1:**
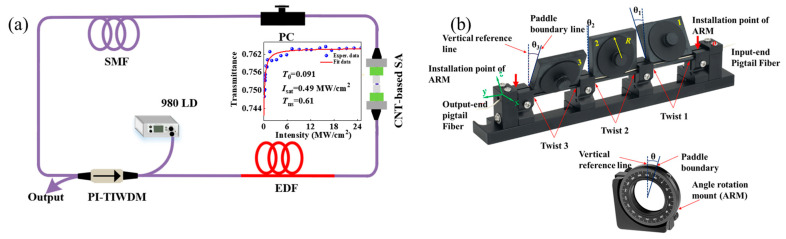
(**a**) Experimental setup of tunable wavelength-multiplexed-bound dual-frequency fiber laser, inset: saturation absorption curve of SA, (**b**) model of three-paddle PC.

The CNT-based SA is assembled in the tunable wavelength-multiplexed-bound dual-frequency fiber laser for generating mode locking pulses. Figure 1 displays the experimental schematic diagram of the bound dual-frequency fiber laser cavity. A 2.6 m long erbium-doped fiber (EDF) acts as the gain medium, and a 980 nm laser diode (LD) is used to pump the gain medium. The EDF has about 7.2 dB/m peak absorption at 980 nm. The pump power is injected into the fiber laser ring cavity through a fiber hybrid device, i.e., polarization-independent, tap-isolator-wavelength division multiplexer (PI-TIWDM). The fiber-based hybrid device combines the functions of polarization-dependent isolator, 80/20 optical coupler and wavelength division multiplexer. The polarization controller (PC) is used to tune the pulse states in cavity. A home-made CNT-SA is fabricated by the method of optical deposition with 0.86 mg/mL single-wall CNT solution, and displays 65% transmission at 1560 nm region. The CNT-SA is deposited on an FC/APC ferrule and is connected to the fiber cavity. The nonlinear saturation absorption characteristics of the SA were measured using a dual-detector method. A home-made femtosecond fiber laser based on nonlinear polarization rotation technology was employed as a test light source. The femtosecond fiber laser has a central wavelength of 1561 nm, a pulse width of 112 fs, and a repetition rate of 21 MHz. The saturation absorption curve of SA is plotted in the inset of Figure 1a. The measured modulation depth, saturated optical intensity, and unsaturated absorption loss are 0.091, 0.49 MW/cm^2^, and 0.61, respectively. The Hi1060 pigtail fiber length of PI-TIWDM in the cavity is about 0.5 m, and its dispersion is about 5.5 ps/nm/km. The dispersion of EDF at 1550 nm is about −18.5 ps/nm/km. The single-mode fiber (SMF) used in the laser cavity is about 6.1 m, and its dispersion is about 15.9 ps/nm/km. The total cavity group velocity dispersion is calculated as −0.078 ps^2^, and the fiber laser used in our experiments operates in the anomalous dispersion region. The 20% energy of pulse is extracted and monitored by a spectrum analyzer (Anritsu, MS9710C, Kanagawa Prefecture, Japan) with 0.05 nm maximum spectral resolution, a 5-GHz bandwidth photodetector, a frequency spectrograph (KEYSIGHT, N9320B, made in Santa Rosa, CA, USA), a 4-GHz, 20-GS/s oscilloscope (KEYSIGHT, DSO99404A, made in Santa Rosa, CA, USA), and an autocorrelator (AC, APE SM1600, made in Berlin, Germany). With the vertical direction as the reference line, the initial deflection angles *θ* of the three wheels in the PC are shown in Figure 1b. The positive directions of *x* and *y* represent the slow axis and fast axis directions, respectively. The initial angles of the three wheels were 19°, 2°, and −23°, respectively, where the negative value indicates the corresponding wheel rotated along the -*x* axis starting from the *z*-axis direction. The rotation angle is defined as the angle between the reference line and boundary of the paddle, and the boundary of the paddle refers to the paddle surface near the -*x*-axis direction. To record the paddle’s rotation angle, two angle rotation mounts are mounted at both ends of the PC. The rotation angle indicated by the angle dial represents the PC’s rotation angle *θ*.

## 3. Results

**Figure 2:**
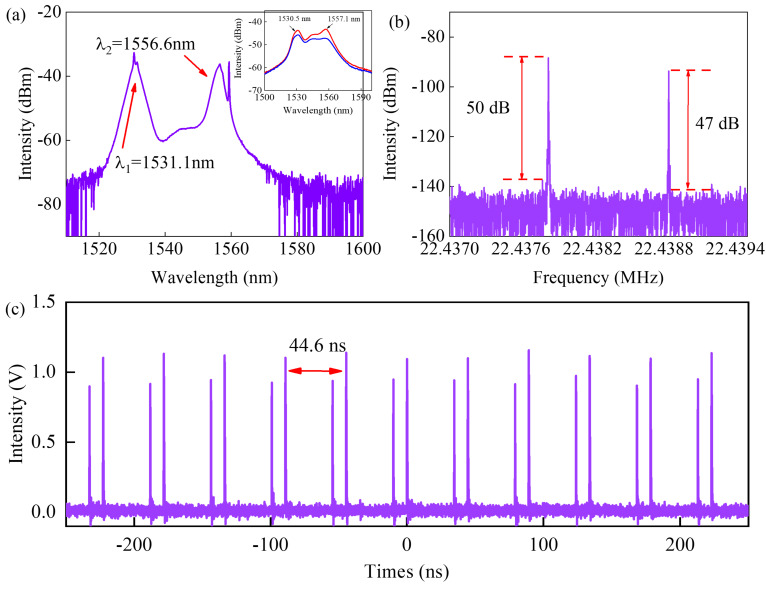
Dual-frequency pulse characterization. (**a**) Output spectrum, inset: gain spectra measured in fiber laser under different PC states, (**b**) radio frequency, (**c**) temporal pulse train.

When the pump power is increased to 89 mW, the fiber laser cavity generates the dual-frequency pulse. The typical output characterizations of dual-frequency pulses are shown in Figure 2. The output spectrum shows two center wavelengths in Figure 2a, which are located at λ_1_ = 1531.1 nm and λ_2_ = 1556.6 nm. The 3 dB bandwidth of them is about 2.03 nm. The radio frequency spectrum of the output pulse exhibits two distinct repetition frequencies, which are 22.4378296 MHz and 22.4388381 MHz, as shown in Figure 2b. The repetition frequency difference calculated from the experimental data is Δ*f*_r_ = 1.01 kHz. According to the Ref. [21], the relation of $\Delta f_{r}=f_{r}^{2}\beta_{2}L\left(\lambda_{2}-\lambda_{1}\right)$ can be used to illustrate the effect of center wavelength difference on the repletion frequency difference. Based on the laser parameters in the experiment, the theoretically calculated frequency difference Δ*f*_r_ is 1.001 kHz. The experimental results are consistent with the theoretical results. Where *β*_2_*L* is the total net dispersion in cavity, *f*_r_ is the repetition frequency of the mode-locked pulses. The signal to noise rate difference of the two frequencies is 3 dB. Figure 2c shows a temporal sequence of the two asynchronous pulses in a 500 ns range, with a 44.6 ns time interval between two adjacent pulses with the same repetition frequency. To investigate the generation mechanism of dual-wavelength pulses, we tested the gain spectrum in a fiber laser under pump of 25 mW, as shown in the inset in Figure 2a. We observe that the gain spectra display two peaks located at 1530.5 nm and 1557.1 nm, respectively. Changing the polarization states in cavity, the gain coefficient at two wavelengths will vary. With suitable polarization state, the peak at 1557.1 nm could disappear. Therefore, lasers at the two wavelengths could experience varied gains with polarization state, which the model has been employed in previous works [16,22]. Additionally, by altering the polarization state, the linear birefringence and cavity loss within the laser cavity can be controlled, and the characteristic can be treated as an equivalent Lyot filter [23]. Since the inherent birefringence of single-mode fiber is relatively small, the generation of wavelength-multiplexed pulses results from the combined effects of the dual gain peak of EDF and the equivalent Lyot filter, with the former playing a dominant role. The average output power of the dual-frequency pulse is about 2.1 mW.

**Figure 3:**
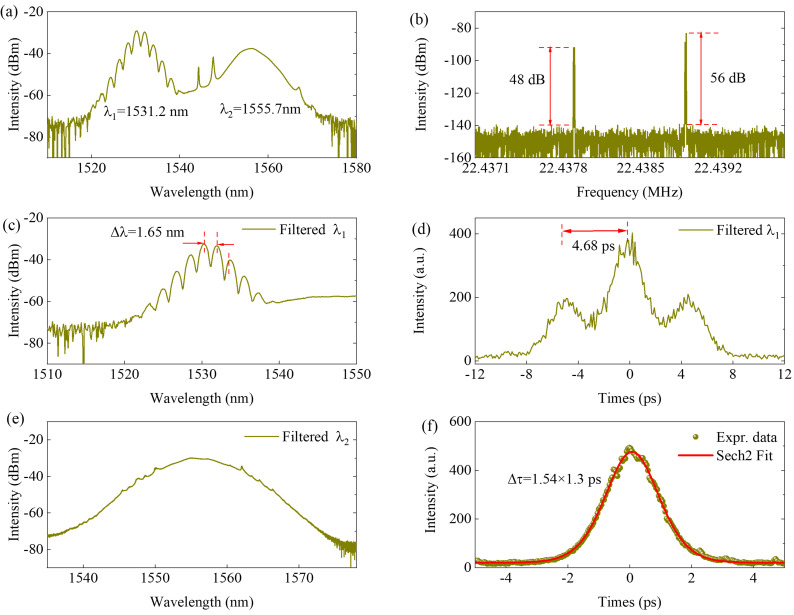
Dual-frequency pulses with bound state located at 1531.2 nm. (**a**) Output spectrum, (**b**) radio frequency spectrum, filtered (**c**) spectrum and (**d**) AC curves of λ_1_, filtered (**e**) spectrum and (**f**) AC curves of λ_2_.

Increasing pump power to 95 mW, a dual-frequency bound pulse is achieved, which one of the two asynchronous pulses evolves into the bound state under suitable polarization states. When the polarization rotation angle of PC is set as 46°, one of the asynchronous pulses located at λ_1_ = 1531.2 nm evolves into a bound soliton, as shown in Figure 3. From Figure 3a, we can note that the output spectrum of λ_1_ = 1531.2 nm exhibits periodical modulation. The 3 dB bandwidth of λ_2_ soliton spectrum increases to 6.47 nm. The repetition difference between the two asynchronous pulses is −8 dB, as shown in Figure 3b. In order to study the features of dual-frequency bound solitons, a 1530 nm coarse wavelength division multiplexer [24] is used to extract one of the two pulses, in which the bandwidth and channel spacing are 13.5 nm and 20 nm, respectively. The spectra of two asynchronous pulses are shown in Figure 3c and Figure 3e, respectively. The fringe spacing in Figure 3c is about 1.65 nm. The AC trace of the λ_1_ pulse is shown in Figure 3d, in which the curve exhibits three peaks, and the height ratio between the peaks is 1:2:1, which means that the two bound pulses have the same intensities. The separation between the two solitons is about 4.68 ps, in accordance with the fringe spacing in Figure 3c. The AC trace of the λ_2_ soliton is shown in Figure 3f, and the pulse width is about 1.3 ps, which is fitted by sech^2^ fitting. The average output power of the dual-frequency pulse is about 1.9 mW.

**Figure 4:**
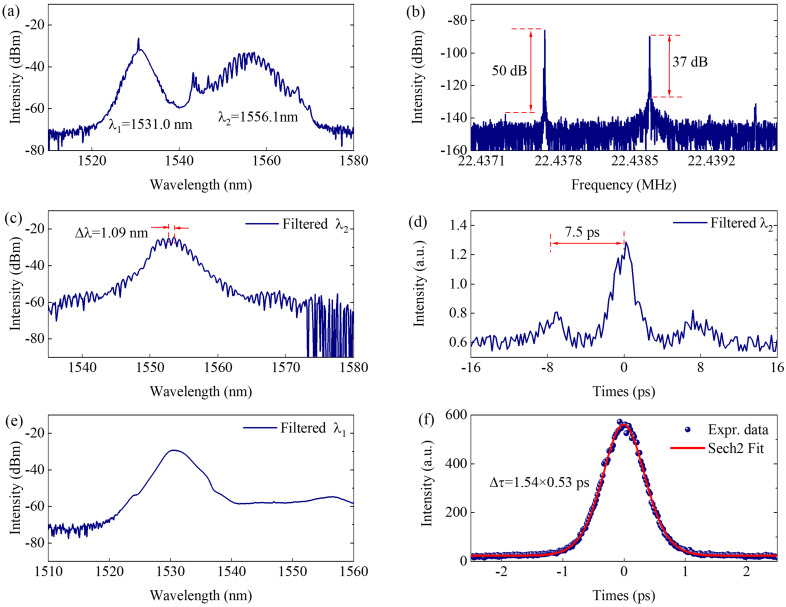
Dual-frequency pulses with bound state located at 1556.1 nm. (**a**) Output spectrum, (**b**) radio frequency spectrum, filtered (**c**) spectrum and (**d**) AC curves of λ_2_, filtered (**e**) spectrum and (**f**) AC curves of λ_1_.

Keeping the pump power of 95 mW, one of the asynchronous pulses located at λ_2_ = 1556.1 nm is switched into a bound state when the polarization rotation angle of PC is set as 67°, as shown in Figure 4. The λ_2_ soliton spectrum exhibits small periodical modulation, while the λ_1_ soliton spectrum shows an obvious continuous wave in the spectral peak, which can be seen from Figure 4a. The repetition difference between the two asynchronous pulses is increased to 13 dB, as shown in Figure 4b. The filtered spectra of the two pulses are shown in Figure 4c and Figure 4e, respectively. The fringe spacing in Figure 4c is about 1.09 nm. The AC trace of the λ_2_ pulse shown in Figure 4d exhibits three peaks, and the temporal interval between the two bound solitons is 7.5 ps. It can be seen that the height ratio between the three peaks in AC is about 2:7:2. This abnormal height ratio can be illustrated using the intensity difference between the two solitons in the bound state [25]. The AC trace of λ_1_ soliton shown in Figure 4f shows the pulse width is about 0.53 ps by sech^2^ fitting. Compared with the results in Figure 4, the bound pulses show lower coherence characteristics and stability. In experiments, we should carefully adjust the PC states to obtain this state. This state can only remain stable for about 20 min. Once this stability is lost, the fiber laser will step into a conventional dual-frequency pulse state, such as the state shown in Figure 2. The average output power of the dual-frequency pulse is about 1.5 mW. As mentioned above, the dual-peak gain spectra of EDF can be regarded as a polarization-dependent spectral filter within the cavity. In the soliton region, linear group-velocity dispersion, nonlinear phase accumulation through self-phase modulation, and amplitude modulation produced by SA are the key factors affecting pulse generation. During the switching of bound state pulse, the pulse energy quantization and polarization-dependent spectral filtering provides the conditions. With suitable polarization states, the net gain (loss) at 1530.5 nm is higher (lower) than that at 1557.1 nm, and the energy of pulses at 1530.5 nm reaches a certain value and exceeds 0.1 nJ; the nonlinear phase accumulation can not be balanced by group-velocity dispersion, the pulse will split into a bound state and achieve a new balanced state. In this process, SA is needed to promote the pulse’s formation and stability. Therefore, the pulse at a wavelength with higher relative gain will be inclined to generate bound solitons under suitable conditions such as pump power and polarization state.

**Figure 5:**
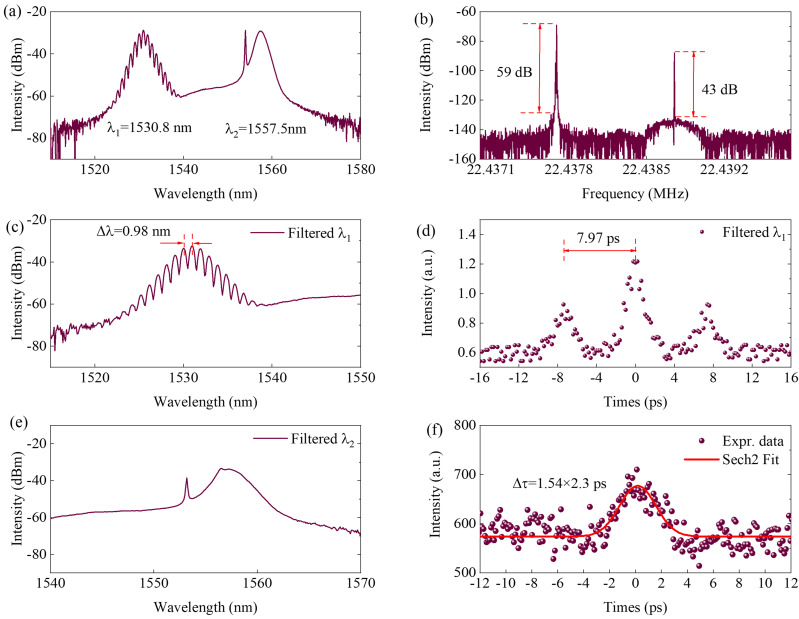
Dual-frequency pulses with bound state located at 1530.8 nm. (**a**) Output spectrum, (**b**) radio frequency spectrum, filtered (**c**) spectrum and (**d**) AC curves of λ_1_, filtered (**e**) spectrum and (**f**) AC curves of λ_2_.

Continuing to change the polarization rotation angle in the same direction to 132°, one of the asynchronous pulses located at λ_1_ = 1530.8 nm is switched into the bound state, as shown in Figure 5. From Figure 5a, we can see that the λ_1_ soliton spectrum exhibits periodical modulation. The relative phase between the two bound solitons at λ_1_ is about 0. It is different from that of in Figure 3a, which the relative phase is about π. In this case, the repetition frequency difference increases to 1.1 kHz, and the signal to noise ration increases to 16 dB, which can be seen in Figure 5b. After filtering, the spectral interferometry of soliton molecule shows a 0.98 nm spectral fringe period, and the soliton separation is about 7.97 ps, as shown in Figure 5c,d. The corresponding output spectrum and AC trace of λ_2_ soliton are shown in Figure 5e,f, and the AC trace shows the pulse width is about 2.3 ps by sech^2^ fitting. The average output power of the dual-frequency pulse is about 1.1 mW. Because the autocorrelator used in our experiments is equipped with a PMT detector, the AC trace of a low-energy pulse could also be measured. The minimum average output power of the filtered pulse is ~0.42 mW.

## 4. Numerical Simulation

In order to gain a deeper understanding of the experimental results, the split-step Fourier method is applied to solve the coupled Ginzburg–Landau equations. These equations account for dispersion, nonlinearity, birefringence, gain, and loss effects [26],

(1) $$\begin{array}{l} \frac{\partial u_{x}}{\partial z}=-i\frac{\Delta \beta }{2}u_{x}+\delta \frac{\partial u_{x}}{\partial t}-i\frac{\beta_{2}}{2}\frac{\partial^{2}u_{x}}{\partial t^{2}}+i\gamma \left({\left|u_{x}\right|}^{2}+\frac{2}{3}{\left|u_{y}\right|}^{2}\right)u_{x}+\frac{g}{2}u_{x}+\frac{g}{2\Omega_{g}^{2}}\frac{\partial^{2}u_{x}}{\partial t^{2}}\\ \frac{\partial u_{y}}{\partial z}=i\frac{\Delta \beta }{2}u_{y}-\delta \frac{\partial u_{y}}{\partial t}-i\frac{\beta_{2}}{2}\frac{\partial^{2}u_{y}}{\partial t^{2}}+i\gamma \left({\left|u_{y}\right|}^{2}+\frac{2}{3}{\left|u_{x}\right|}^{2}\right)u_{y}+\frac{g}{2}u_{y}+\frac{g}{2\Omega_{g}^{2}}\frac{\partial^{2}u_{y}}{\partial t^{2}} \end{array}$$

where *u_x_* and *u_y_* indicate the orthogonal components of the pulse, which propagate along the fast and slow axes of the fiber, respectively. *z* and *t* are the propagation distance of pulse in fiber, the retarded time, respectively. *β*_2_ and *γ* correspond to the fiber parameters, i.e., second-order dispersion coefficient and third-order nonlinear coefficient, respectively. Δ*β* and 2*δ* are the wave number difference and the inverse group velocity difference between the *u_x_* and *u_y_* components, respectively. The bandwidth of the EDF is governed by Ω*_g_*, and its saturable gain can be expressed by $g=g_{0}\exp\left(-E_{p}/E_{s}\right)$, which is a function of the small-signal gain coefficient *g*_0_, pulse energy *E*_p_, and gain saturation energy *E*_s_.

**Figure 6:**
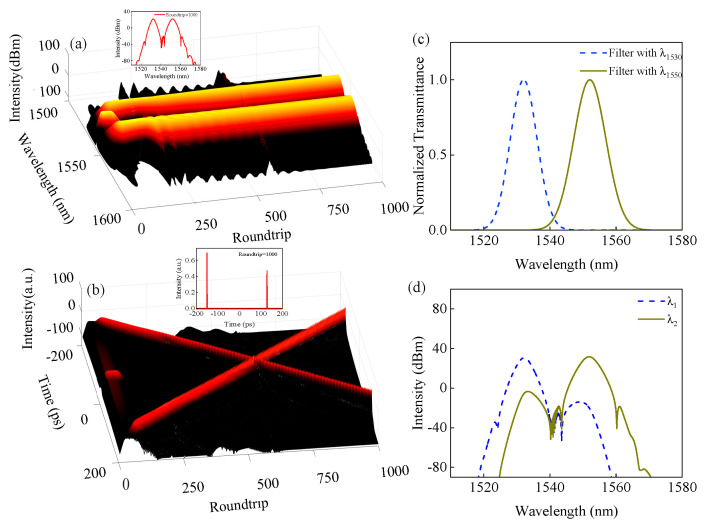
Simulation results of dual-frequency pulses. (**a**) Evolution of output spectra, (**b**) evolution of temporal pulses, (**c**) two filter spectra and (**d**) corresponding filtered spectra.

In our simulations, the uneven gain profile of EDF is considered, and the two uneven gain peaks of EDF are 1530.5 nm and 1557.1 nm, respectively. The initial input signal is a sech-type pulse with a 1ps pulse width and peak intensity of 2 × 10^−10^ W. The parameters are carefully matched to experimental conditions: Ω*_g_* = 25 nm, *g*_0_ = 1.38 m^−1^, and *E*_s_ = 20 pJ. For the 2.6 m EDF, *γ* = 1.3 W^−1^ km^−1^ and the dispersion parameter is −18.51 ps/nm/km, and for the 6.1 m SMF, *γ* = 1.3 W^−1^ km^−1^ and the dispersion parameter is 17 ps/nm/km. For the 0.5 m Hi1060, *γ* = 1.3 W^−1^ km^−1^ and the dispersion parameter is 5.503 ps/nm/km. For the CNT-based SA, Δ*T* = 0.09, *I*_sat_ = 0.5 MW cm^−2^, and *T*_ns_ = 0.61. When the rotation angle is set as 15° in the simulation, the stable wavelength-multiplexed dual-frequency pulse occurs after 1000 roundtrips, as shown in Figure 6. From Figure 6a,b, we can see that the dual-wavelength pulse generates at roundtrips = 35, and the time separation between two pulses first decreases and then increases, and a collision occurs at roundtrips = 595. The insets in Figure 6a,b are the output spectrum and temporal pulses at roundtrip = 1000, respectively. Two Gauss-type filters are used to split the two pulses, and the filter parameters of center wavelength and bandwidth are 1530 nm, 5.8 nm and 1550 nm, 6.8 nm, respectively, as shown in Figure 6c. The filtered spectra are shown in Figure 6d.

**Figure 7:**
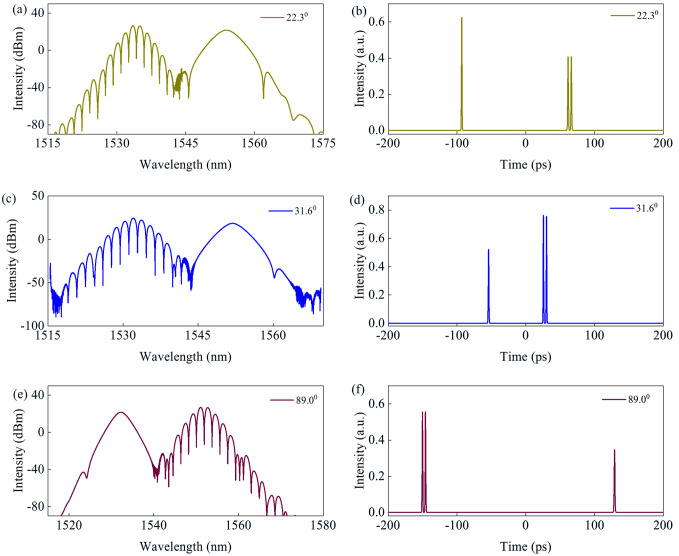
Simulation results of dual-frequency bound pulses. (**a**,**c**,**e**) Output spectra, and (**b**,**d**,**f**) temporal pulses under different polarization rotation angles.

**Figure 8:**
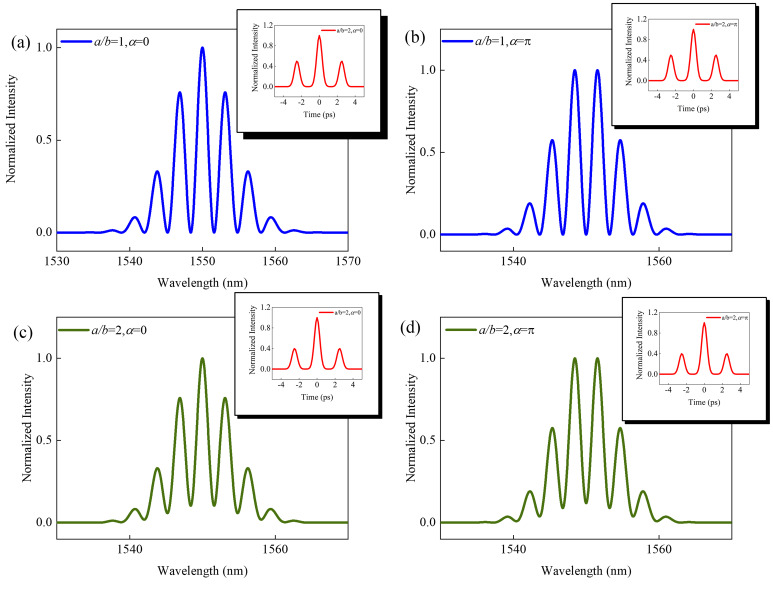
Simulation results. Interference spectra and AC traces of two bound-state solitons with (**a**) *a*/*b* = 1, *α* = 0, (**b**) *a*/*b* = 1, *α* = π, (**c**) *a*/*b* = 2, *α* = 0, (**d**) *a*/*b* = 2, *α* = π.

When the E_s_ = 20 pJ and rotation angle is set as 22.3°, one of the asynchronous pulses located at λ_1_ is switched into the bound state. The spectrum exhibits a π relative phase of fringe pattern at λ_1_, as shown in Figure 7a,b. When the rotation angle is set as 31.6°, the relative phase between the two bound solitons at λ_1_ of spectrum changes to 0°, as shown in Figure 7c,d. Unlike the experiment’s tuning rotation angles significantly, the bound soliton located at λ_2_ occurs with angle of 89°, as shown in Figure 7e,f. Different from Figure 4, the height ratio between the three peaks is about 1:2:1, and the spectral modulation depth is larger than that of in Figure 4. Considering the two bound solitons with a τ temporal separation and α relative phase, the time domain pulses can be described as $a*f\left(t\right)+b*g\left(t-\tau \right)\exp\left(i\alpha \right)$, where *f*(*t*) and *g*(*t*) denote the complex amplitudes of two solitons. The spectral intensity can be expressed as [27],

(2) $$|AF\left(\omega \right)+BG\left(\omega \right)\exp\left(-i\left(\omega t-\alpha \right)\right){|}^{2}=A^{2}F^{2}\left(\omega \right)+B^{2}G^{2}\left(\omega \right)+2ABF\left(\omega \right)G\left(\omega \right)cos\left(\omega \tau -\alpha \right)$$

where *F*(*ω*) and *G*(*ω*) denote the Fourier transform of *f*(*t*) and *g*(*t*). From express (2), we note that the spectral modulation depth varies along with the spectral intensities and pulse envelope, i.e., pulse peak intensities and pulse widths. To better illustrate the effect of the relative phase between two solitons on the interference fringes in the interference spectrum, we now proceed with a numerical analysis, and assume that the two bound solitons are $A(t)=a*\sec h\left(t/T_{0}\right)+b*\sec h\left(\left(t-\tau \right)/T\right)\exp\left(i\alpha \right)$, where the *T*_0_ is pulse width. In the simulations, *T*_0_ = 0.5 ps, amplitude ratio *a*/*b* = 1 or 2, *α* = 0 or π. As shown in Figure 8a,b, when the relative phase is 0, the interference spectrum exhibits characteristics of a main peak and side lobes, while at a relative phase of π, the interference peaks are symmetrical about the spectral center. When the amplitude ratio is 1, the amplitude ratio of the main lobe to the two side lobes in the AC curve is approximately 1:2:1. When the amplitude ratio increases to 2, the intensity ratio in the autocorrelation curve is approximately 2:5:2, as shown in Figure 8c,d. The simulated results and experimental data exhibit essentially identical spectral and autocorrelation curve characteristics. Therefore, the abnormal height ratio of three peaks in AC trace in experimental results can be explained by different peak intensities between the two pulses.

## 5. Discussion

We experimentally and theoretically investigated the generation and control characteristics of wavelength-multiplexed dual-frequency bound-state soliton pulses in a CNT-based bound-dual-frequency fiber laser. By adjusting the pump power to 89 mW, dual-frequency pulses located at ~1531.1 nm and 1556.6 nm are obtained. Because of two uneven gain peaks of EDF and polarization-dependent loss, one of the asynchronous pulses located at λ_1_ or λ_2_ is switched into the bound state, and exhibits different spectral interferograms and modulation depths, as shown in Figure 3, Figure 4 and Figure 5. For the bound solitons centered at λ_1_, the relative phase between the two bound solitons can be switched from 0° to 180°. For the bound solitons centered at λ_2_, the two bound solitons own different peak intensities, leading to larger intensity difference in AC trace and a small spectral modulation depth in output spectrum between the main peak and side peaks. Coupled Ginzburg–Landau equations have been used to give deep insight into the physics mechanisms behind the experimental results, and the numerical simulation results matches well with our experimental observations. These results will provide a new way to understand multiple soliton interaction and has practical application value in the field of optical frequency combs generation.

## Data Availability

The data presented in this study are available on request from the corresponding author.
